# Misinterpretation of a Gunshot Injury by a Non-forensic Medicine Expert: A Case Report of Misguidance in a Criminal Investigation Involving the Unusual Firearm Injury 'Kronlein Shot’

**DOI:** 10.7759/cureus.53724

**Published:** 2024-02-06

**Authors:** Toshal Wankhade, Binay Kumar

**Affiliations:** 1 Forensic Medicine, All India Institute of Medical Sciences, Patna, Patna, IND; 2 Forensic Medicine & Toxicology, All India Institute of Medical Sciences, Patna, Patna, IND

**Keywords:** digital forensic evidence, crime scene, kronlein shot, gun shot wound, forensic medicine expert

## Abstract

In India, the majority of postmortem examinations are conducted by medical professionals who lack expertise in the field of forensic medicine. Medicolegal autopsy services that are done by forensic experts are primarily confined to tertiary care centers, although a significant portion of medicolegal postmortem examinations occurs in non-tertiary healthcare setups. In this context, postmortem examination reports occasionally fail to encompass essential medicolegal features, resulting in dissatisfaction among both crime investigators and the deceased's relatives. As the doctors managing these postmortem examinations are not experts in the field of forensic medicine, if a case involves an unusual pattern of injury, the situation becomes further complicated. The absence of proper forensic assessments heightens the risk of the crime investigation heading in the wrong direction.

Firearm injuries are well known for their varied pattern of injury. The present case reports one of the unusual patterns of firearm injury where a gunshot injury with close contact on the head resulted in the bursting of the skull, which is commonly referred to as a Kronlein shot. Such injuries involve extreme skull mutilation. Unlike typical contact gunshot injuries, in cases of extensive facial and cranial destruction, locating the entry wound and detecting other typical features of firearm injury become challenging. This complexity confused the autopsy surgeons of this case who were not experts in forensic medicine and mis-framed the opinion regarding the causative weapon which in turn confused the police official in the crime investigation. The case was later on referred to our institute for forensic medicine expert opinion and opinion was given after consideration of postmortem examination findings mentioned in the postmortem report, photographs of the deceased, circumstantial evidence gathered by police, and correlating all these facts with standard published literature.

The case underscores the essential role of forensic experts in decoding complex medicolegal mysteries and ensuring accurate justice delivery. The case also highlights the importance of the need for comprehensive forensic examinations and considering circumstantial evidence in drawing various conclusions in a medicolegal autopsy.

## Introduction

Postmortem examination in India done at non-tertiary level health care centers is usually carried out by medical officers who are not experts in forensic medicine. Many times, the autopsy surgeon fails to interpret important features and evidence while performing postmortem examination, which may mislead the crime investigation. There are multiple instances where the investigating agency and also relatives of the deceased are not satisfied with the diagnosis given by the autopsy surgeon. In many cases, dead bodies have been sent for a second autopsy at a tertiary care center for a second opinion from the forensic pathologist [1,2].

There are many areas in medicolegal autopsy wherein thorough knowledge of forensic medicine is required to draw various conclusions that play a crucial role in crime investigation and delivery of proper justice. In developing countries, the quality of autopsy is always questioned in court [3]. The court highly relies on the postmortem examination report for delivery of justice. Hence, the postmortem report should be accurate and should be done by an expert. In some cases where the injury patterns are unusual, the situation becomes further complicated when autopsy surgeons are not experts in forensic medicine. 

Firearm injuries are known for variations in the injury pattern. In typical firearm injury, we might have penetrating wounds (when the bullet enters the body but does not exit) or we might have perforating wounds (when the bullet enters and exits the body). The entry wound is usually characterized by the presence of an abrasion collar, tattooing on the skin, muzzle impression of the gun (when the muzzle is put in close contact with the body), and the effect of fire or smoke particles on the affected tissue. However, not all gunshot injuries show a typical injury pattern on the tissue involved. These features vary from a case-to-case basis and depend on many factors like the range of fired bullets, type of firearm used, type of ammunition used, etc. Multiple patterns and variations of wound ballistic are very well documented in the literature [4]. In penetrating injury, the bullet may be recovered from the body cavity; however, in this case, we have only an entrance wound. An autopsy surgeon should have a thorough knowledge of the various injury patterns of firearm injury cases to frame proper opinion while performing a medicolegal autopsy.

Kronlein shot is one of such atypical injuries where the entire skull is burst and mutilated including evisceration of the brain [5]. The present case describes how the Kronlein shot can confuse the autopsy surgeon particularly those who are not experts in forensic medicine in drawing the conclusion of a causative weapon which can mislead the investigation of crime. 

## Case presentation

**Figure 1:**
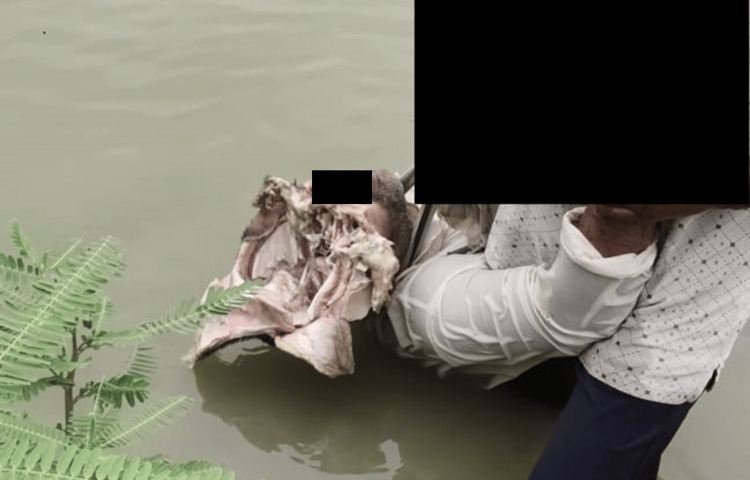
Body recovered from a shallow lake.

In this case, a dead body was found lying in a shallow lake in a village. When nearby villagers noticed it, they informed the case to the police (Figure 1). The police took the dead body to a nearby district hospital to perform an autopsy. Autopsy was performed by medical officers who were not experts in forensic medicine. The diagnosis made by the medical officer was death due to head injury and the causative object was given as a hard and blunt weapon along with a heavy sharp weapon. The relatives of the deceased were claiming it as an alleged assault. However, during the investigation, police got some circumstantial evidence indicating the case was suicide and they also found a firearm weapon at the crime scene. However, the opinion given by the autopsy surgeon did not support their investigation. The opinion of the medical officer indicated the manner of death was more of a homicide. Hence, the case was referred to our department for forensic medicine expert opinion as police had doubts regarding the manner of death and also the weapon used.

For framing the opinion in this case, we were provided a postmortem report prepared by the concerned medical officers, photographs of the dead body and the crime scene investigation, and also the circumstantial evidence gathered by police regarding the case during the course of enquiry.

Findings from postmortem examination

The postmortem report of the deceased has revealed the following key findings: the deceased had comminuted fractures of all skull and facial bones. Both eye orbits were missing. Overlying scalp skin showed incised-looking margins. Internal examination of the thoracic and abdominal cavity showed that all internal organs were intact and pale. No mechanical injuries were mentioned in the postmortem examination other than the facial and skull area. The cause of death was mentioned as ‘Death due to head injury’. Opinion regarding the causative object was mentioned as ‘Injury with a sharp weapon associated with a heavy hard and blunt object’.

Findings from photographs of the dead body provided by the police

**Figure 2:**
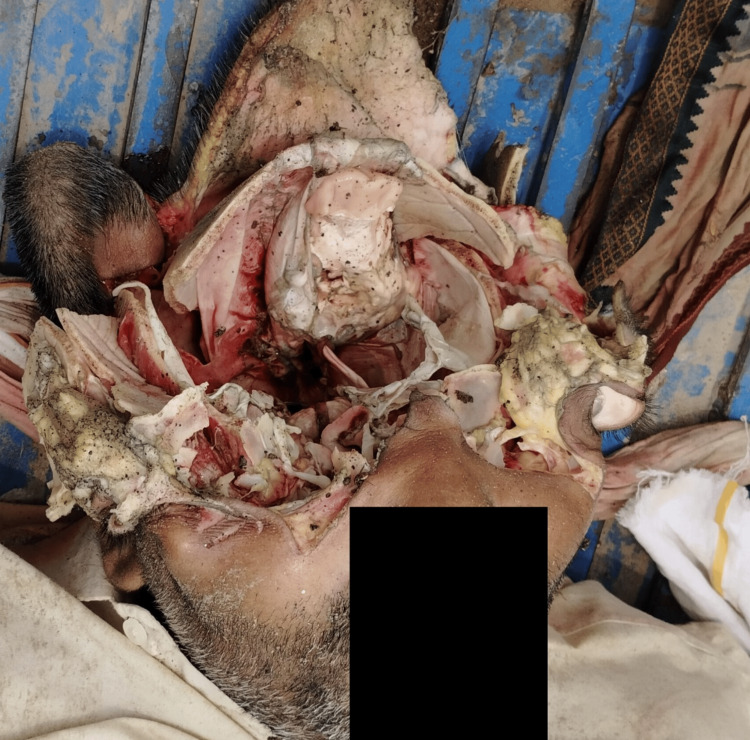
Burst of the skull and surrounding tissue.

**Figure 3:**
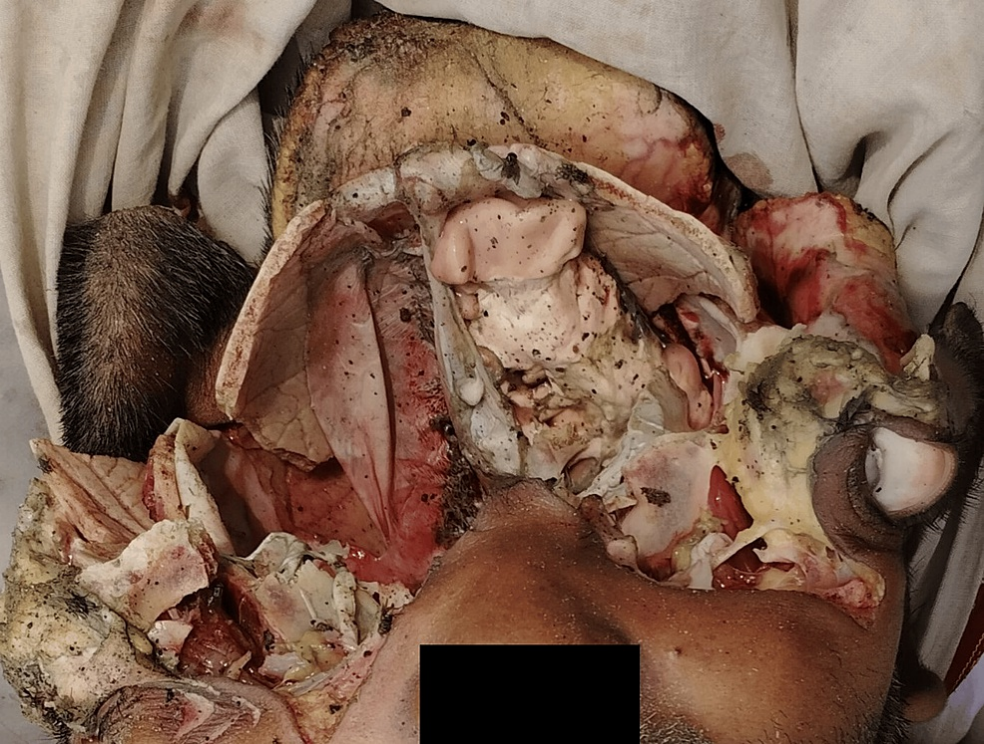
Empty cranial cavity.

Careful examination of photographs of the dead body provided by the police revealed the following: the entire skull (except the occipital area) and upper part of the face involving both orbits were burst open, with comminuted fracture of craniofacial bones & tears of the overlying skin, tissues, and the meninges in a radial manner in all directions. The cranial cavity of the deceased was widely opened, and the brain was missing in the cranial cavity (Figure 2). The surrounding skin was disrupted and showed split lacerations (Figure 3). From the external appearance, no entry and exit wound could be found as the skull was completely mutilated. There were multiple dark-colored spots present inside the cranial cavity, but these spots cannot be recognized as gunpowder residue as the body was merged in shallow water and hence multiple mud particles may be present inside the cavity. There were no other injury marks noticed below the line of fracture and only the skull cavity was burst. Clothes showed red-colored stains resembling the staining due to blood.

Circumstantial evidence gathered by the police 

**Figure 4:**
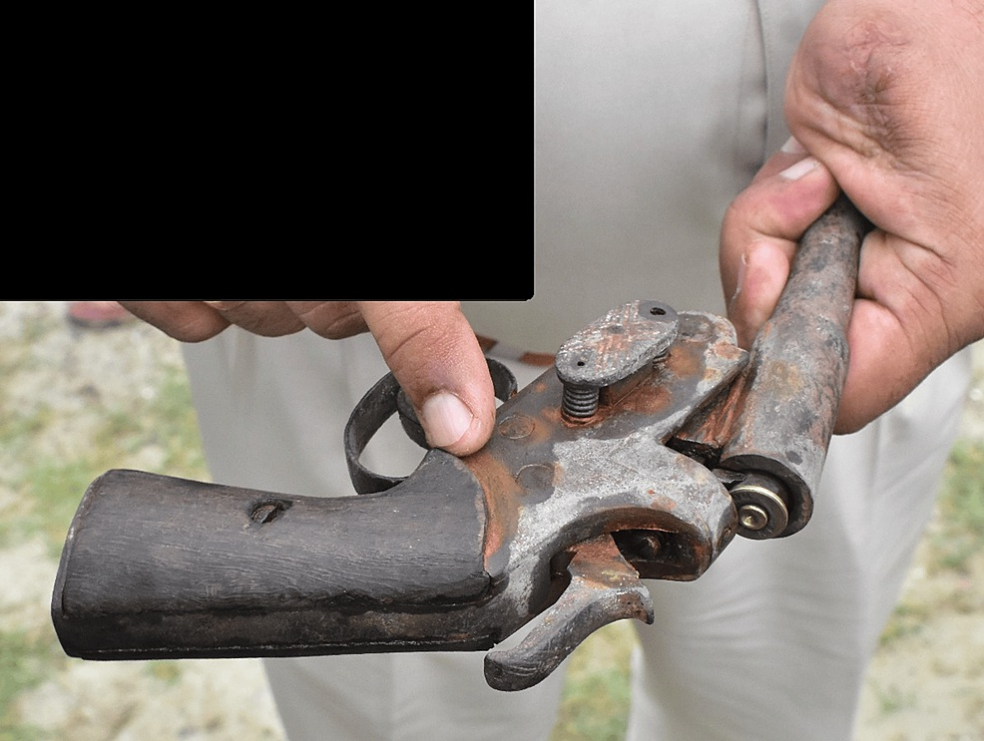
Indigenous iron-made pistol (desi katta) recovered from the lake where the body was lying.

When the crime scene was done by the police in the presence of a magistrate, they recovered an indigenous iron-made firearm weapon (desi katta) in the lake from where the dead body was recovered. The chamber of the barrel of the weapon had an empty cartridge case of .315 bore-fired bullet (Figure 4).

The police also investigated Google search history from the mobile of the deceased. The Google search history of the deceased was provided to us by police in hard copy. The search history reveals that prior to death the deceased was searching for methods of suicide. In his search history, he was looking for information on the easiest methods of death, how someone feels before dying, and the effects of a bullet when it hits the brain or heart. The searches were conducted in the Hindi language.

On investigation of the CCTV footage of the nearby area, the police found that the deceased was moving alone in the early morning toward the lake.

## Discussion

Kronlein shot or bursting of the skull occurs when a bullet is fired from a firearm weapon within a low range (contact) on the head. In such a shot, when the bullet along with gasses passes through the close skull cavity, it imparts momentum to the tissues directed radially outward from the bullet’s path. This causes sudden compression inside brain tissue and a wave of tremendous pressure develops inside the cranial cavity. As brain tissue is compressed, very high pressure is exerted on cranial bones and a sudden burst of the skull may take place. The brain may be eviscerated out of the cranial cavity. Orbits may blow out. In such injuries, typical entry and exit wounds cannot be appreciated [5-10]. 

A contact gunshot injury refers to a wound inflicted by a firearm in which the muzzle of the gun is in close contact with the body at the time of discharge. Usually, when a contact shot occurs, it shows a muzzle impression when a weapon has hard contact on the head; in loose contact, a muzzle impression may not be present. Typical features of contact shot are bullet entry wound and exit wound if the bullet exited. Skin margins are irregular and a stellate-shaped opening may be seen at the entry wound. There is evidence of suit particles, tattooing, and other typical features of firearm injury from which the autopsy surgeon gives an opinion regarding the causative object [5-10]. These peculiar features are not seen in the Kronlein shot when the skull is totally burst, and framing opinion becomes a little complicated as these are rare phenomena. 

The injury pattern of a Kronlein shot or bursting of the head due to a firearm has been widely published in various literature studies. The so-called Kronlein shot was first described by ‘Rudolph Ulrich Krönlein’ who was a Swiss surgeon [7]. In one of his articles, he reported a case of suicide by gunshot where the skull was burst, the cranial cavity was empty, and the brain was eviscerated out of the skull cavity and was found lying near the dead body.

Pathak reported a case where he received a referred case from a medical officer in which the skull of the deceased exploded; initially, it was thought as a fatal road accident by the police and medical officer but after autopsy at the tertiary center, it was found to be a case of gunshot injury by a country-made firearm. He proposed that the mechanism of this injury was like a Kronlein shot [11].

Cullen and Luckasevic reported a case of suicide where the skull was blasted due to a contact wound by a homemade shotgun [12]. The brain was eviscerated.

In all the above-cited literature, the Kronlein shot is well described with its features and mechanism of causation. Bursting of the head is a well-documented feature that may mimic head injury due to hard and blunt objects or due to explosives. The injury pattern observed in the present case i.e. the wide burst of the head, missing brain from the cranial cavity, and blown of orbits indicates the Kronlein shot-like mechanism.

To support it as a case of firearm there is circumstantial evidence of recovery of firearm weapon from the site where the body was recovered. On careful observation of photographs, it was noted that below the fracture line, no other external injury was present. If it had been wound by a heavy sharp weapon or hard and blunt weapon as earlier opined by a medical officer there should have been additional injuries like abrasion, laceration, or contusion below the fracture line, but the wound was radially distributed all around the cranial cavity. Furthermore, no other injury marks are mentioned in postmortem reports like struggle signs which are commonly a feature of homicidal death.

In an earlier opinion given by a medical officer regarding causative weapons as a ‘hard and blunt weapon along with the heavy sharp weapon,’ it appears impossible for the manner of death as suicidal because bursting of the head with heavy sharp or with a hard and blunt object is just impossible by self-infliction. Considering the causative object as opined by the medical officer most appropriate manner of cause of death can be homicidal death. However, circumstantial evidence gathered by the police like his Google search history as described above and CCTV footage where the deceased moved out of his house toward the lake in the early morning gave rise to suspicion that the case was a suicide. Furthermore, the police recovered the firearm weapon from the site. The main query to police officials was whether the injuries mentioned in the postmortem examination and evident in photographs were possible by the firearm weapon. For this reason, they referred the case to our institute for expert forensic medicine opinion.

By considering the various findings mentioned in postmortem examination reports, photographs of the deceased dead body, circumstantial evidence gathered by police, and correlating all facts with available literature we framed opinion as 'Injuries mentioned in postmortem report & observed in photographs supplied by a police officer are possible with a contact shot by firearm weapon.'

With this opinion, the possibility of suicide cannot be conclusively ruled out in this case, as circumstantial evidence points toward suicide. However, the initial opinion indicated the most possible manner of death as a homicide.

The Google search history recovered by the police from the deceased's phone is crucial circumstantial evidence that raised suspicions regarding the manner of death as suicidal. This highlights the importance of digital forensic investigation in medicolegal autopsy. Digital forensics is a branch of forensic science that involves the collection, analysis, and preservation of electronic evidence, such as data from computers, smartphones, and other digital devices, to investigate and prevent cybercrime or traditional crimes with a digital component [13].

It is said that every human activity leaves a digital trace [14]. There are multiple instances where digital data are used to solve the crime in a court of law [15]. 

In the context of inquests, the expertise of forensic specialists becomes particularly crucial. Their ability to navigate through intricate injury patterns, interpret crime scene evidence, and integrate technological advancements, contributes significantly to investigating the cause of death accurately. Forensic experts play a crucial role by offering valuable opinions that might escape individuals lacking specialized training in the field.

One of the studies by Kumar et al. emphasized the need for forensic specialists in the inquiry into the cause of death process. This study highlights that the involvement of experts significantly improves the accuracy and reliability of conclusions drawn during inquests [16].

## Conclusions

The observed injury pattern in this case is of a Kronlein shot. Thus here, we presented a case of an atypical and not often seen firearm injury in forensic practice which a non-forensic medicine expert wrongly interpreted.

The autopsy surgeon, when drawing conclusions, must be vigilant about recognizing unusual injury patterns. Overlooking them can lead to incorrect conclusions and potentially misguide the crime investigation. In the present case, if the injury had been misinterpreted as caused by a hard or blunt object, or a heavy sharp weapon, as per the initial opinion, it could have incorrectly suggested a homicidal manner of death. Thus, drawing conclusions without adequate evidence or investigation can adversely impact the justice delivery system.

The case emphasizes the importance of a comprehensive forensic medicine opinion and highlights the necessity of a multidisciplinary approach for a thorough forensic examination through analysis of postmortem findings, photographs, and circumstantial evidence. Moreover, the integration of digital forensics in this context highlights the evolving nature of forensic investigations and the importance of considering all available sources of evidence.

The case underscores the importance of a forensic medicine expert's opinion in the detection of complex medicolegal mysteries. It emphasizes the need for experts in the field of medicolegal autopsy, and in the absence of such specialists, autopsy-performing medical professionals should receive adequate training in forensic medicine.
